# CVD and Oxidative Stress

**DOI:** 10.3390/jcm6020022

**Published:** 2017-02-20

**Authors:** Karla Cervantes Gracia, Daniel Llanas-Cornejo, Holger Husi

**Affiliations:** Institute of Cardiovascular and Medical Sciences, University of Glasgow, BHF Glasgow Cardiovascular Research Centre, 126 University Place, Glasgow G12 8TA, UK; karly.cgracia@gmail.com (K.C.G.); llanasdaniel@gmail.com (D.L.-C.)

**Keywords:** cardiovascular disease, CVD, oxidative stress, reactive oxygen species

## Abstract

Nowadays, it is known that oxidative stress plays at least two roles within the cell, the generation of cellular damage and the involvement in several signaling pathways in its balanced normal state. So far, a substantial amount of time and effort has been expended in the search for a clear link between cardiovascular disease (CVD) and the effects of oxidative stress. Here, we present an overview of the different sources and types of reactive oxygen species in CVD, highlight the relationship between CVD and oxidative stress and discuss the most prominent molecules that play an important role in CVD pathophysiology. Details are given regarding common pharmacological treatments used for cardiovascular distress and how some of them are acting upon ROS-related pathways and molecules. Novel therapies, recently proposed ROS biomarkers, as well as future challenges in the field are addressed. It is apparent that the search for a better understanding of how ROS are contributing to the pathophysiology of CVD is far from over, and new approaches and more suitable biomarkers are needed for the latter to be accomplished.

## 1. Introduction

In 1985, the term oxidative stress was coined by Sies [1] to broadly describe a disturbance in the balance of reactive oxygen species (ROS) and antioxidants. However, its definition has been changing over the years due to the wide variety of outcomes it can produce. Currently, oxidative stress is defined as an event where a transient or permanent perturbation in the ROS balance-state generates physiological consequences within the cell, for which the precise outcome depends on ROS targets and concentrations.

Besides cellular damage, ROS have also been shown to be involved as messengers in signaling pathways in a balanced-“normal”-state. In a homeostatic living system, ROS concentrations fluctuate in a controlled manner and are preserved through antioxidants and other enzymes [2]. Once this homeostatic state starts to fail and ROS levels cannot be controlled, oxidative stress becomes apparent. Guanylate cyclase activation, nervous system physiology regulation, immune cell differentiation and response regulation [3,4,5,6], as well as the mediation of phosphatases, kinases, growth factor signaling pathways and even stem cell differentiation [7,8,9] are some of the mechanisms that have been reported to be influenced by ROS, producing either beneficial or damaging consequences.

Xenobiotics, such as radiation, drugs, habits like smoking, as well as environmental agents, interact with cellular sources of ROS, inducing its generation. Mitochondria, endoplasmic reticulum, peroxisomes and enzyme systems, involving for example NADP oxidases and xanthine oxidase, are some of the intracellular ROS sources. Additionally, ROS action varies according to different cell types and depending on its source, type, location, concentration and target, thereby leading to a variety of physiological or pathological consequences [10,11]. Moreover, there are different types of ROS, such as superoxide (^−^O_2_), hydroxyl radicals (HO), hydrogen peroxide (H_2_O_2_), singlet oxygen (O_2_), peroxynitrite (OONO^−^) and nitric oxide (NO) [11]. Overwhelming quantities of ROS are involved in several pathologies, such as cancer, neurodegenerative diseases, diabetes and cardiovascular diseases (CVD), due to its role promoting inflammation, damaging DNA and proteins, as well as lipid peroxidation [12]. In this review, the main focus will be CVD and the influence that oxidative stress has on these pathologies.

## 2. CVD Highlights and Their Relationship with Oxidative Stress

CVD are multifactorial disorders and, according to the World Health Organization (WHO), are the leading causes of death worldwide [13]. CVD represents 31% of deaths globally in 2013, causing approximately 17.5 million deaths per year. The 2016 CVD statistics update by the American Heart Association (AHA) reported that about one in three persons in the U.S. was affected with one or more CVD types in 2013 [14], which has also an economic impact. Globally, its costs are constantly rising, with an estimate in 2010 of $863 billion and an expectation of $1044 billion by 2030. The basis of CVD encompasses damage and remodelling of blood vessels that can result in blood flow restrictions affecting the heart and nervous system. There are several disorders that comprise CVD, namely coronary artery disease (CAD), stroke, hypertension, heart failure, rheumatic aetiologies, congenital heart disease and peripheral vascular disease. In 2011, the AHA included preeclampsia (PE) as a risk factor of CVD. Within the CVD spectrum, other well-known risk factors, such as obesity, diabetes, tobacco smoking, a sedentary and unhealthy lifestyle, family history, genetic predisposition and oxidative stress, also play an important role in CVD. Aging is another risk factor, though a non-modifiable one, since it increases CVD prevalence mainly due to the accumulation of oxidative damage [15].

The main leading cause of CVD is atherosclerosis. It is the hardening and narrowing of arteries that consequently reduces the flow and delivery of blood and oxygen throughout the body. It is characterized by plaque formation in the inner coronary artery walls, consisting of bulks of LDL cholesterol, cellular waste and surrounding materials [16]. Atherosclerotic plaques are caused by molecular changes induced by cytokines, hormones, growth factors and oxidative species, mainly due to the interaction between endothelial cells, LDLs and macrophages. Endocytosis of oxidized LDLs occurs within macrophages, an event that is quite slow and, therefore, can lead to an accumulation in the intima resulting in the development of atherosclerosis [17].

LDLs can be chemically modified in several different ways. Native LDL molecules are susceptible to be oxidized (oxLDL) [18], glycated (gLDL) [19], acetylated (acLDL) [20], ethylated [21] and methylated [21]. Studies regarding endothelial cell injury caused by modification on LDLs have pointed to an association of gLDL and oxLDL with the initiation of atherogenic processes [22]. Nowadays, it is clear that changes in LDL and its components play an important role in the initiation and generation of atherogenic effects, especially when these modifications occur within cells of the arterial wall. Modified components of native LDLs, such as apoB (LDL surface protein), which mediates the ability of LDL to bind to its receptor, have been the subject of studies in order to elucidate the relationship of these types of modification and the triggering of the atherogenic processes [23].

A non-enzymatic reaction oversees the glycation of lipoproteins in arterial walls. The extent of this modification is dependent on the glucose concentrations and the exposure time of native non-modified LDLs [24]. Non-enzymatic glycation of LDLs can occur in all patients, but subjects with diabetes mellitus can present more severe effects. Glycation of LDL mainly occurs in lysine residues of apoB that are located in an LDL receptor-binding domain. This change causes the loss of electropositive charges on the modified LDL, decreasing its affinity to its receptor, thus increasing its lifetime in plasma [25]. All of this was shown to enhance the uptake of gLDLs by human aortic cells [26] and macrophages [27], promoting the accumulation of cholesteryl esters and atherosclerosis. An additional atherogenic effect of LDL glycation is that the modified molecules become more susceptible to be oxidized, creating a double modified native LDL (gly/oxLDL), which might have a greater atherogenic potential in comparison with gLDL [28].

**Figure 1:**
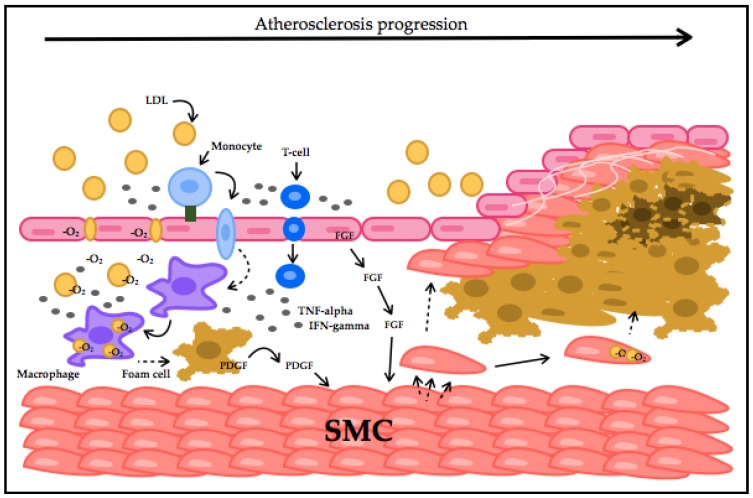
Atherosclerotic plaque formation process. LDLs at a high concentration inhibit endothelial cells’ (EC) endocytosis capacity, migrate and accumulate in the intima. LDLs get oxidized and induce VCAM (green square) expression in EC. Monocytes are recruited into the intima by VCAM interaction. Monocytes transform into macrophages, which take-up oxLDLs, forming foam cells. Macrophages and EC secrete chemokines and recruit T-cells. T-cells produce TNF-alpha and IFN-gamma, amplifying inflammation. Fibroblast growth factor (FGF) and platelet-derived growth factor (PDGF) stimulate smooth-muscle cells (SMC) migration and proliferation. SMCs can also accumulate lipids, migrate and proliferate. A lipid core is generated with necrotic foam cells surrounded by SMCs and a collagen fibrous cap, resulting in thrombus formation.

In early stages of atherogenesis, LDLs are oxidized (oxLDL). Once oxLDLs starts to accumulate, the disease progresses. oxLDL activates the endothelium by the production of adhesion molecules, which recruit monocytes and T-cells, considered a key stimulator for the immune system response [17]. Once adhesion of inflammatory cells to endothelial cells takes place, migration into the arterial intima follows. Here, monocytes differentiate into macrophages that induce atherosclerosis progression by promoting internalization of oxLDL and, along with T-cells, release pro-inflammatory cytokines and ROS to keep oxidizing LDLs [29]. This leads to smooth-muscle cell migration into the intima, followed by proliferation. This contributes to the formation of an atherosclerotic plaque by apoptosis or foam cell formation [30]. Atherosclerosis progresses and CVD genesis starts by accumulation and conversion of these molecules into potentially damaging forms, (Figure 1). As mentioned above, damaging ROS species have shown a strong association with the vascular endothelium. The ROS influence on endothelial underlying molecules that can promote apoptosis, necrosis and therefore thrombosis of atherosclerotic plaques makes oxidative stress a crucial hallmark of CVD and is defined as its early causative factor [31,32].

## 3. ROS Types and Principal Sources in CVD

Among the ROS that have been associated with CVD induction or progression are ^−^O_2_, H_2_O_2_, OONO^−^ and HO. ^−^O_2_ and H_2_O_2_ are ROS produced enzymatically and have been shown to be involved in physiological signaling and pathologies. Furthermore, ^−^O_2_ can be converted spontaneously by superoxide dismutase (SOD) into H_2_O_2_ [33].

On the other hand, OONO^−^ and HO are not considered ROS signaling molecules. These ROS molecules are known to be highly reactive and contribute to oxidative stress and tissue damage. HO is formed by H_2_O_2_ catalysis, and glutathione can scavenge it. ^−^O_2_ and NO reaction produce OONO^−^, which causes severe damage in endothelial tissue due to NO depletion and uncoupling of the endothelium nitric oxide synthase (eNOS), leading to a cycled reaction that promotes dysfunction progression in the endothelium [34].

**Figure 2:**
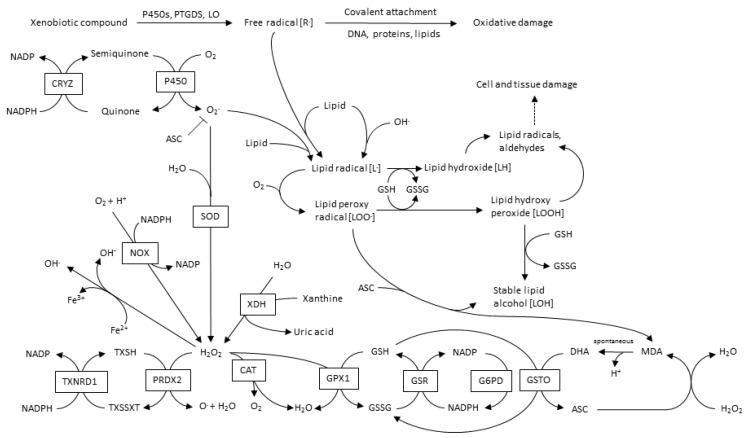
Metabolic pathways in ROS production and metabolism.

The main sources of CVD-ROS are mitochondrial NADPH oxidases (NOX), xanthine oxidases (XO), lypoxidases (LO) and myeloperoxidases (MPO). Furthermore, ROS-source-crosstalk has been well documented, where H_2_O_2_ can activate NOX, as well as induce xanthine dehydrogenase transformation into XO; OONO^−^ induce ^−^O_2_ production; and even more interesting, mitochondria and NOX interact with each other, leading to an oxidative cycle [35]. A global overview of ROS and its metabolic pathways is shown in Figure 2.

### 3.1. Mitochondrial-ROS 

Since 1961, ROS production has been shown to occur within mitochondria [36] linked to the respiratory chain [37]. Mitochondria are nowadays considered as the major source of ROS in most cells, particularly in cardiac cells [31]. The most abundant damaging ROS in vascular tissue is ^−^O_2_, and ROS-^−^O_2_ generation within mitochondria results mainly from the electron transport chain (ETC) pathway, although it has also been linked with H_2_O_2_ production [37]. Mitochondrial-ROS (mtROS) is involved in several pathways and has a role in the innate immune response as a bactericidal effector [38].

There is a close relation between mtROS and endothelial dysfunction, and an evident cross-talk between mtROS and NOX-ROS has been demonstrated in several studies [39,40,41,42,43] where the production of one molecule leads to the induction of the other and vice versa. This mechanism is directly associated with endothelial dysfunction through the influence of mtROS and ^−^O_2_ production stimulation in endothelial cells (EC). In this tissue, NO is considered fundamental to maintain its normal functionality; however, in the presence of ^−^O_2_, NO concentration is decreased, and the reaction between those two molecules produces the extremely reactive species OONO^−^ that can generate irreversible cellular damage. A damaging cycle also takes place here, leading, due to the oxidation of the endothelial nitric oxide synthase (eNOS) cofactor BH_4_ by OONO^−^, to eNOS uncoupling, and instead of producing NO, induce an increase in ^−^O_2_ production [32,44]. Other studies have also confirmed eNOS uncoupling consequences through inactivating phosphorylation, thereby demonstrating the association between ROS sources, functionality and production [45,46,47].

The location of NOX4 in the outer mitochondrial membrane is another factor of interest in mitochondrial crosstalk and NOX-ROS production. NOX4-ROS has been reported to be involved in the activation of mitochondrial ROS generation by inner mitochondrial channels, such as ATP-sensitive potassium channels (K_ATP_), as well as the permeability transcription pore (mPTP), from which mtROS can be transported out of the mitochondria to the cytosol, thereby maintaining NOX4-ROS production and the cross-talk cycle [40]. Moreover, studies of disorders, such as diabetes, hypertension and aging have shown an association between NOXs and mitochondria [48,49].

Several studies have shown that mitochondria and NOX both predominate as major ROS sources in diabetes and that hyperglycemia is associated with mitochondria-NOX ROS crosstalk [50]. Type 2 diabetes has a causal link with CVD, and several studies of insulin secretagogues, such as sulfonylureas in CVD, have shown a link to K_ATP_ channels. Their opening inhibition, as well as over-induction lead to vascular complications and even to sudden cardiac death. In a diabetic state, a predominantly decreased function of K_ATP_ channels has been observed, and it is suggested that, due to inhibited ischemic preconditioning, myocardial infarction can happen. This can lead to a dramatic change of the K_ATP_ channels status, which might abruptly induce its opening [51]. This event suggests a regulation of mitochondrial-NOX interaction [32].

Within mitochondria, two sources of ROS production predominate in the ETC, complex I (CI) and complex III (CIII), which can stimulate the opening of pores (mPTP and K_ATP_) located in the inner and outer mitochondrial membrane (IMM and OMM) [34]. A recent study determined that CIII-ROS has cardio-protective functions, while CI-ROS production results in damage and association with CVD [52]. mPTP opening was found to be correlated with incremental ROS production. ROS generated from complex III delays channel opening, and its presence has been related with functional recovery improvement after ischemic reperfusion (I/R) injury from which insult has been characterized as a potent risk factor of oxidative stress. CI-ROS is not considered as a dominant source of ROS under normal or healthy conditions, but it has been shown to be the major mitochondrial source of electron leakage in I/R hearts [53]. Previous studies have also associated mPTP opening and cardio-protective effects in I/R injuries from mitochondrial systems involved in ROS production with deleterious consequences. mPTP inhibition by different molecules, such as cyclosporine A (CsA) and inorganic phosphate, confer protection [54,55]. Furthermore, inhibition of other mitochondrial systems that are involved in the production of ROS, such as monoamine oxidase (MAO) or p66^Shc^, have been associated with protection against I/R injury, heart failure and vascular injury [56,57,58,59]. Although many studies have shown new insights into the role of mt-ROS in CVD, there is still a substantial lack of knowledge of the detailed mechanisms involved in mt-ROS production, as well as the mode of action of the complexes.

### 3.2. NADPH Oxidases 

**Figure 3:**
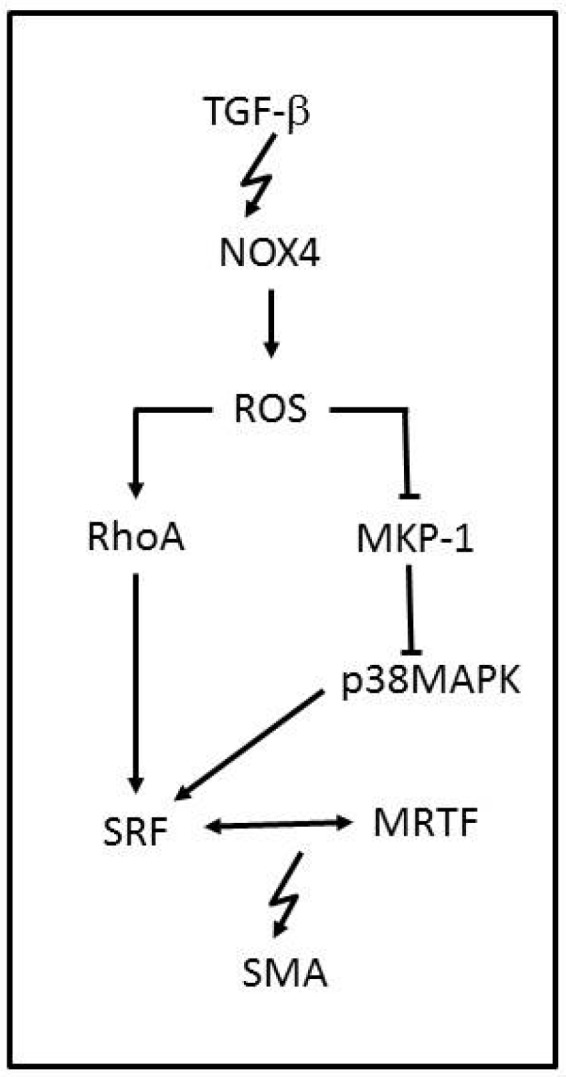
NADPH oxidase-modulated pathway in vascular smooth muscle cell differentiation. The TGF-β-mediated induction of NOX4 causes an inhibition of MKP-1 and activation of RhoA by ROS. Under physiological conditions, MKP-1 inhibits p38MAPK, thereby preventing the RhoA-mediated release and association of transcription elements SRF, which can be activated by p38MAPK, and MRTF, which drive SMA gene expression.

NOX family enzymes are also known to be major contributors of ROS production. NOX1, NOX2, NOX4 and NOX5 are complexes that assemble in the membrane and are expressed in vascular tissue with the apparent only purpose of producing ROS. NOX2 and NOX4 have been reported to produce ROS in cardiomyocytes and fibroblasts and NOX1, NOX4 and NOX5 in the vascular smooth-muscle cells (VSMCs). NOX enzymes are characterized by ^−^O_2_ production, but NOX4 is the exception to the rule due to its predominant H_2_O_2_ generation. NOX-ROS molecules are involved in physiological signaling, antimicrobial and inflammation host defence pathways and cellular differentiation [60,61]. NOX4 is known to be involved in VSMCs, stem cells, fibroblasts and cardiac cell migration and differentiation [62], and a link between NOX4-ROS and the regulation of cellular phenotype was reported [63]. NOX4 has been shown to increase ROS production while induced by transforming growth factor-β (TGF-β) (Figure 3). Once NOX4 is expressed, it regulates smooth-muscle α-actin (SMA), by oxidating the MKP-1phosphatase that can inactivate p38MAPK. ROS can also activate RhoA, which is a kinase that regulates the release of myocardin-related transcription factor (MRTF) and the activation of the serum response factor (SRF), leading to SMA activation. Therefore, when NOX4-ROS inhibits MKP-1, p38MAPK phosphorylates SRF, which binds to MRTF, and activates SMA, inducing VSMCs differentiation. NOX4 function is associated with vascular injury. However it is known to respond in later stages, counting as a susceptibility factor for advanced injury [64]. Although many pathological events have been associated with an increased function of NOX4, it has a protective role in hypertension disorders [65]. NOX4 overexpression has been shown to reduce basal blood pressure, which is linked to H_2_O_2_ production.

**Figure 4:**
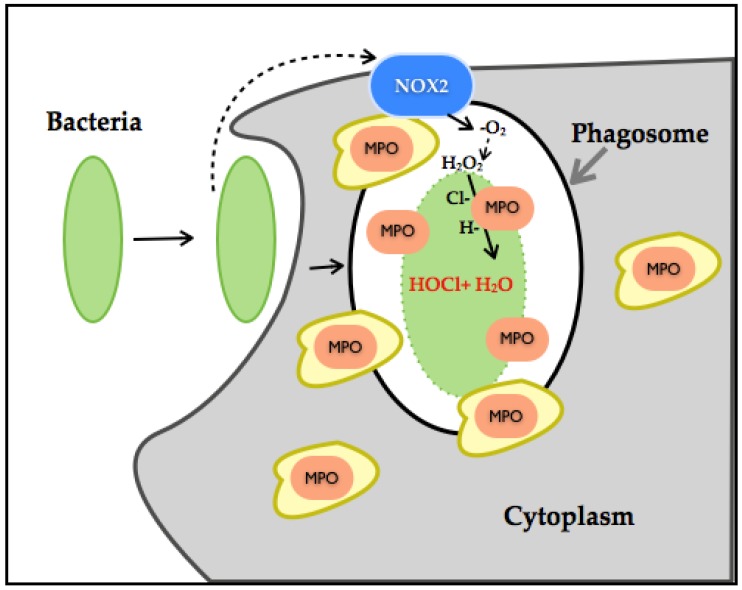
Illustration of neutrophil NOX-HOCl production. Neutrophils have phagosomes and granules in its cytoplasm. Granules contain myeloperoxidase (MPO) (orange). The NOX2 complex (blue) gets assembled once the neutrophils initiate bacterial endocytosis. NOX2 produces H_2_O_2_ by dismutation, and MPO reacts with H_2_O_2_ and chloride ions, thereby generating HOCl and water, leading to neutrophil bactericidal effects.

A recent study has established the dual role of NOX-ROS [66]. This work showed that H_2_O_2_ generated through NOX disrupts tyrosine phosphorylation signaling by thiol oxidation of p-Tyrosine, leading to its conversion to DOPA protein. DOPA protein can promote the disruption of networks and therefore can lead to an alteration of biological processes. As such, in the presence of bacteria, H_2_O_2_ interferes with bacterial tyrosine kinase signaling by decreasing its virulent damage (Figure 4).

However, thiol-oxidation is a considerable pathway modifier event. When a protein undergoes DOPA modification under oxidative stress or increased generation of H_2_O_2_, it can disrupt biological processes and lead to disease. H_2_O_2_ production has also been linked to MPO, which are also ROS-producing enzymes. Neutrophil-NOX production has been identified as a powerful bactericidal through molecular oxygen reduction to ^−^O_2_, its dismutation to H_2_O_2_, culminating with chloride ions in the generation of hypochlorous acid (HOCL). HOCL even at low concentration is considered as extremely reactive, inducing irreversible oxidation modification of methionine, and through this mechanism, it is considered an efficient bacterial killer system. In conjunction with high NOX-H_2_O_2_ production, it can alter entire systems through degradation or misfolding of proteins [67,68,69,70,71]. H_2_O_2_ is also associated with the activation of the p66^SHC^ protein, which is involved in apoptosis and ROS metabolism. H_2_O_2_ influences the p66^SHC^ serine phosphorylation state and therefore its internalization and accumulation in mitochondria. p66^SHC^ binds to cytochrome C and promotes H_2_O_2_ generation and therefore apoptosis [72]. Another noteworthy aspect is that NOX1 and NOX2 function in a molecular complex and therefore depend on activators, regulators and binding proteins (p22^Phox^, NoxO1, NoxA1, Rac1/2 for NOX1 and p22^Phox^, p47^Phox^, p67^Phox^, p40^Phox^ and Rac1/2). NOX5 is dependent on calcium and therefore regulated by calcium stimuli, which adds a considerable amount of complexity in its role in CVD. On the other hand, NOX4 remains constitutively active in the presence of O_2_ within the cell, depending on other proteins not for its activation, but for stability and localization. NOX4 is also located within the mitochondria, specifically in the OMM, which demonstrates its role as a major ROS source [73,74,75].

Upregulation or disturbance of NOX enzymes has been studied and shown to be associated with endothelial dysfunction, atherosclerotic plaque formation, LDL oxidation, macrophages and neutrophil recruitment, an increment in the pro-inflammatory molecules’ expression, senescence and apoptosis in vascular tissue, contributing to CVD genesis and progression [76,77,78].

Risk factors, such as UV radiation, smoking, high calorie diets, diabetes, hypoxia, and endogenous factors, such as pro-inflammatory molecules, growth factors, platelet-derived growth factor (PDGF), and hormones, such as Ang II [76], insulin [77], can trigger NOX production. Since all of the NOX-ROS trigger factors are associated with endothelial dysfunction, it can easily interact with other ROS sources and these with each other, producing a ‘never ending’ oxidative stress state [78,79].

Cardiovascular dysfunction, as well as atherosclerosis has been related with an increased expression of NOX2, NOX4 and NOX5, but only at early stages [80,81,82]. However, NOXs functionality was shown to differ between age groups. NOX1/2 are mostly influential in young mice, and inhibition by the deletion of p47phox in ApoE^−/−^ mice reduced ROS levels and attenuated the development of atherosclerosis [83,84,85]. A recent report showed that aged ApoE^−/−^ and ApoE^−/−^ /p47phox^−/−^ mice that developed atherosclerosis and vascular dysfunction were characterized by mitochondrial dysfunction related with an upregulated expression of NOX4 in VSMCs [86]. To add to the general complexity, diabetes has also been closely linked with CVD and NOX activity. NOX1 is activated by a diabetic state, where hyperglycemia induces AngII and PDGF production that leads to vascular dysfunction and NOX1-ROS production, hypertension and atherosclerosis [87]. Moreover, high glucose has been reported to increase cardiomyocyte NOX4 production, which leads to cardiac damage [88].

Other CVD phenotypes, such as pulmonary artery hypertension (PAH) and atrial fibrillation (AF), have been related with NOX activity. An upregulation of NOX2 and NOX4 is found in PAH, and their inhibition was demonstrated to reverse PAH in animal models [89,90]. Atrial fibrillation (AT) has been associated with an excess of ROS and a decrement of NO in myocardial tissue, and NOXs-ROS are suggested to be involved in the induction phase of AF development [91]. This study also showed that mitochondria and uncoupled ROS generation by eNOS influence long-term damage in AF.

### 3.3. Xanthine Oxidase

Hyperuricemia, defined as measurable elevated levels of uric acid (UA), is a common factor observed in CVD patients. The involvement of UA in CVD pathophysiology has still to be elucidated, though xanthine oxidoreductase, the enzyme responsible for UA production, was suggested to play an important role in CVD genesis. Xanthine dehydrogenase (XDH) and xanthine oxidase (XO) reduce hypoxanthine and xanthine to UA. However, XDH uses NAD+ as an electron receptor and, when triggered by factors, such as hypoxia, cell apoptosis, inflammation or ROS generation from other sources (such as mitochondria or NOX) is converted to XO, whereby oxygen is used as an acceptor of electrons, which results in the generation of ^−^O_2_ and H_2_O_2_ [92]. XO was the first biological mechanism to be identified as a producer of ROS [93] and, apart from its pathological function, has been identified physiologically as a biological system that confers protection from pathogen infections. However, XO-ROS production has been linked to endothelial dysfunction mainly due to the reaction of ^−^O_2_ and NO that produces the OONO^−^ reactive oxygen radical responsible for cellular damage [94]. Additionally, an excess of UA in certain CVD states, such as congestive heart failure (CHF) and CAD, can function as a marker of upregulated XO activity, and inhibition through allopurinol therapy only showed an improvement in patients with this characteristic [95,96,97,98].

A modulated XO activity has also been related with pulmonary hypertension [99], and the signaling pathway of the transcription factor early growth response-1 (Egr-1) has targets implicated in proliferation, inflammation and fibrosis; and it is known to be involved in pulmonary vascular remodelling that leads to pulmonary hypertension [100]. Egr-1 itself was shown to be inducible by XO-ROS through a chain of events initiated by ROS, whereby ROS interacts with the VSMCs-EGFR membrane receptor, leading to MAPK/ERK1/2 phosphorylation, Egr-1 downstream activation and ultimately to vascular remodelling and CVD [101,102].

### 3.4. Lipoxygenase 

Among the lipoxygenases (LO), 5-LO and 12/15-LO have been shown to display a high correlation with CVD genesis. Atherosclerosis, as well as I/R injury and therefore myocardial infarction have been studied as a function of LO activity, and a tight association between LO enzymatic action, metabolites and the development of CVD itself has been noticed.

Arachidonic acid (AA) is the LO substrate involved in its pathogenic role. AA is oxidized by LO, resulting in hydroperoxides, which are further reduced into hydroxides and leukotrienes. Each LO generates different metabolites according to the carbons in AA in which they act [103]. LOs mediate AngII-induction of NOX in VSMCs, and AA catalysis generates ROS in vascular cells. 5-LO can be upregulated by cytotoxicity and oxidative stress (NOX or mitochondrial ROS), and 5-LO catalysis of AA generates 5-HETE and a leukotriene (LTA4), both of which are lipid mediators [104]. Metabolism of LTA4 by several enzymes produces pro-inflammatory molecules (LTB4, LTC4, LTD4, LTE4) that further interact, communicate and activate cells, such as endothelial cells, macrophages, neutrophils, mast cells, foam cells and T-cells, that subsequently release cytokines and metalloproteinases with pro-atherosclerotic function [105,106,107,108,109]. The involvement of 5-LO in CVD pathogenesis has been postulated due to this modulation of inflammatory cells to increasing chemotaxis, inflammation, permeability and injury by LTA4 [110]. Inhibition of 5-LO was shown to have a beneficial effect in myocardial infarction and ischemia [111], but inhibition of 5-LO products (LTs-LTB4) did not result in a similar improved outcome [112]. It has been highlighted that a much better understanding and dissection of the activity and function of each of the components in the 5-LO pathway is needed in order to develop an effective therapeutic method targeting these molecules [112].

12/15-LO activity is also involved in increased inflammation and oxidative stress and plays an important role in atherosclerosis development [113]. AA metabolites 12-HPETE and 15-HPETE and their reduced HETE forms generated by 12/15-LO are also pro-inflammatory and anti-inflammatory molecules [114], and 12/15-LO overexpression has been associated with a high production of pro-inflammatory cytokines, IL-6 and TNF-alpha [115]. 12/15-LO activity is characterized by oxygenation of LDL molecules [116]. It was also shown that 12/15-LO activity associated with NOX-ROS production plays an important part in oxidative stress in diabetic cardiomyopathy [117]. 12/15-LO displays an increased activity in the diabetic heart. 12/15-LO inhibition has shown a decrement in an oxidative stress marker (4-HNE) in conjunction with ROS generation, and in turn, NOX4 is associated with 12/15-LO activity in diabetic hearts [117]. Since diabetes has been associated with several conditions, such as increased oxidative stress and hypertension, it is conceivable that CVD can be also associated.

Recently, an association between ROS and 15-LO upregulation in pulmonary hypertension (PH) was described in the literature, whereby damage in the pulmonary artery occurs by inducing proliferation, leading to apoptosis [117]. AA-15-LO metabolites (15-HETE) enhance mitochondrial ETC and NOX4-ROS generation and, under hypoxia, further induce endothelial cell migration and pulmonary arterial smooth-muscle cell proliferation by p38 MAPK activation, leading to pulmonary vascular remodelling.

### 3.5. Myeloperoxidase 

Endothelial dysfunction is known to be under the influence of myeloperoxidase (MPO) activity, and its excessive activity can lead to CVD, such as CAD [118,119]. MPO are present in high quantities in neutrophils and to a lower extent in monocytes. When released, MPOs mediate H_2_O_2_ and halide or semihalide ions reaction events that result in the production of hypohalous acids, such as hypochlorous acid (HOCL^−^) and hypothiocyanous acid (HOSCN). These outcomes, generated by MPOs’ activity are believed to have a bactericidal effect. However, when its activity is disturbed or upregulated, a damaging outcome is expected [120]. Several pathologies have been associated with this functional disturbance. HOCL^−^ reacts mainly with nitrogen and sulphur atoms in cysteine residues present in glutathione (GSH). Cys oxidation can inactivate or activate cellular molecules with important roles, such as an inactivation of enzymes by modifying active-site Cys residues or an activation of metalloproteases, such as MMP-7, which leads to a disturbance of the cellular redox balanced state and can initiate atherogenesis [121]. Glutathione sulphonamide is the result of GSH oxidation primarily by HOCL-, which makes it a potential marker for MPO damaged activity [122]. Smoking produces high levels of SCN- and through its reaction with H_2_O_2_ by MPO leads to significantly increased quantities of hypothyocianous acid (HOSCN) [123]. HOSCN is capable of reacting with thiols, resulting in Trp oxidation, and this reaction mainly damages enzymes like tyrosine phosphatases, whereby cells display hyperphosphorylation and altered MAPK signaling, which in turn can culminate in enhanced apoptosis [124]. HOSCN is also able to modify LDL and HDL (oxLDL, oxHDL) [125,126,127] and oxidize NO [128], and the product cyanate reacts with amides and generates a CVD predictor marker [129].

Diverse diseases, such as polycystic ovary syndrome (PCOS) and diabetes, have been related with an increased risk in CVD genesis, and it can be associated with an involvement of inflammation and oxidative stress in its pathogenesis. This is due to a leucocytes recruitment (insulin resistance (IR)), increased ROS production, endothelial cell activation and release of adhesion molecules, leucocyte binding and migration and elevated levels of cytokines [130,131,132]. Leucocyte activity is closely related with this endothelial dysfunction, and it is suggested to have a heavy role in inflammation and CVD risk, especially since MPO is a predominant oxidase within these cells. Recent studies have established a link between the increment of MPO in leucocytes with IR and enhanced ROS production in PCOS patients, thereby highlighting the influence of MPO in oxidative stress events [133,134]. Therefore, MPO has been shown to be a main factor in inflammation and atherogenesis and is an underlying factor of CVD genesis under certain conditions.

## 4. Common Pharmacological Approaches for CVD and Their Relationship with ROS

Current pharmacological approaches for CVD are focused on the use of statins, angiotensin receptor blockers (ARBs) and antiplatelet agents. Although the main goal of these therapies is either to lower blood pressure, regulate lipid content in the bloodstream and to prevent the formation of atherosclerotic plaques, some of them have shown effects that are involved in the production or scavenging of ROS.

Statins are commonly used to treat CVD because of their lipid-lowering action. They do not have a direct influence on ROS; instead, they have an indirect antioxidant effect that inhibits the 3-hydroxy-3-methyl-glutaryl-coenzyme A (HMG CoA) reductase pathway. The inhibition of HMG CoA perturbs the production of farnesyl pyrophosphate and geranylgeranyl pyrophosphate, which are necessary for the generation of O_2_ from NOX. It also has been reported that the use of statins reduces the incidence of CVD through antioxidant properties that enhance the expression of endothelial nitric oxide synthase [135].

The relationship between angiotensin-converting enzyme (ACE) and its effect of increasing the production of angiotensin II is another possible therapeutic option that could revert vascular damage. It has been reported that high levels of angiotensin II contribute to the release of vascular O_2_ [136]. Alternatives have been proposed, and most of them are focused on the inhibition of ACE by using receptor blockers, such as the angiotensin type I receptor blockers, which promote enhancement of plasma levels of angiotensin II to stimulate angiotensin II type II receptors, leading to NO production and vasodilation [137]. In the same way, the widespread use of calcium channel blockers for the treatment of several CVDs due to their antioxidant and antihypertensive effects has also set an opportunity to probe their possible effects over ROS-related pathways [138]. Several studies were analysed in a meta-analysis in patients with CVD that were treated with calcium channel blockers, and it was concluded that the risk of CVD was reduced with the use of calcium channel blockers when compared to the traditional treatments [139].

It is evident that commonly-used therapies have become the first step towards the unveiling of the relationship between drugs and ROS-mediated malignancies, such as CVD. There is still much to confirm, and novel and more specific targeted therapies have appeared in recent years with a positive promise of improving cardiovascular care.

## 5. ROS Biomarkers, Novel Therapies and Challenges

There are several novel potential biomarkers under investigation that can eventually unravel CVD pathological mechanisms to help identify the onset and progression stages and ultimately reduce CVD damage. Table 1 shows several cardiokines that have the potential to be CVD biomarkers [140].

Due to the prevalence of the disease across the global population and the projected incidence rate to double between now and 2050, there is a critical need to develop new treatments that prevent or inhibit CVD risk. It has become very apparent that oxidative stress is the major contributing factor in CVD genesis, and therefore, new therapeutic approaches to combat its effects hold the potential to ameliorate endothelial dysfunction and therefore prevent CVD development.

Caloric restriction (CR) has previously been shown to reduce ^−^O_2_ generation, potentiating NO bioavailability and therefore decreasing endothelial dysfunction [141]. It has been reported that the activation of a member of the sirtuin family of protein deacetylases/deacylases (SIRT1) in old mice by the compound SRT1720 can influence oxidative stress reduction, resulting in a reversal of vascular dysfunction [142]. Furthermore, nicotinamide mononucleotide NMN supplementation is of great interest, since it induces and enhances the activity of SIRT1 by increasing NAD+ bioavailability. NMN was shown to normalize superoxide production and to increase antioxidant MnSOD availability, and it also ameliorates vascular changes that produce arterial stiffness. Interestingly, SIRT3, the mitochondrial form of sirtuins, is known to directly influence MnSOD [143]. Although the exact signaling pathways in which NMN plays a role have not been elucidated yet, its supplementation outcome makes it suitable to be a potential novel therapeutic compound in combatting CVD [144].

Trehalose supplementation has also been studied for whether it can lead to an improvement in microvascular dysfunction and CVD prevention on middle-aged and old adults. It has been reported that trehalose supplementation improves resistance artery endothelium-dependent dilatation (EDD) [145,146]. Since NO mediates this functional aspect, it was implied that trehalose increases NO generation and availability within the cells. However, it was noted that a nutritional alteration had to be factored in with this administration method due to the high caloric content of trehalose-needed doses. It is clear that more research is needed in order for this compound to have a potential application as a novel therapeutic drug [146].

Another example of a potential therapeutic agent is the antioxidant mitoquinone (MitoQ). It has been previously reported that it reduces oxidative stress and increases glutathione peroxidase 1, which is an antioxidant that converts peroxides into alcohol and water [147]. Subsequent research has established the role of MitoQ, where it could be shown to be a modulator of leukocyte-endothelium interaction [148]. MitoQ displayed a protective cardiovascular role by increasing leucocyte velocity and decreasing its flux and adhesion to the endothelium, thus avoiding oxidative stress and inflammation.

Nucleic acid-based therapeutic delivery is also a promising strategy to fight CVD. Viral vectors have been used as DNA-based therapeutics to target endothelial dysfunction. The Kuopio Angiogenesis Trial 301 (KAT301) evaluated the efficacy of a mature form of vascular endothelial growth factor (VEGF-D) using an adenoviral system delivery in CHD patients by NOGA^®^-mediated transendocardial injection. In this study, an increment in myocardial perfusion was detected after three months by VEGF-D influence [149,150]. Although viral vector delivery systems show a promising improvement in endothelial dysfunction and DNA delivery, the possibility to interact with pattern recognition receptors (PRRs) and potentiate endothelial dysfunction accounts for a greater disadvantage. Alternatively, the development of nanotechnological platforms for drug delivery has shown great promise in the treatment and improvement of endothelial dysfunction. An example of the benefits of this system was reported by treating atherosclerotic mice with a nano-emulsion created with omega-3-polyunsaturated fatty acid (PUFA)-rich oil-in-water that selectively delivered 17-B-estradiol into targeted fibrin bulks within the plaque. This system improved NO production, decreased pro-inflammatory molecules and reduced vascular injury, thereby ameliorating atherogenesis [151]. Several nanodelivery systems have been created, among them dendrimers (polymer-based DNA), liposome-DNA complexes, lipid-polymer complexes and hydrogel-graphene oxide complexes. The latter one was trialled by injecting it specifically into infected areas of the myocardium in rodents and showed a decrease in MI injury after disease initiation [152]. Additionally, recent advances in miRNA targeting have also gained attention in treating vascular disorders. Endothelial dysfunction induces a number of genes associated with disease, which make them potential targets that could be silenced by siRNAs. Viral complexes, as well as nanoparticle systems have successfully delivered siRNAs, resulting in silenced molecules, such as CD40 (endothelial activation) and VCAM-1 (adhesion molecule) and, thereby, leading to an improvement in CVD [153,154]. The latest addition in the repertoire of therapeutic options is based on oligonucleotide-based therapies that include antisense oligonucleotides (ASOs), anti-microRNA oligonucleotides (AMOs) and aptamers. ASOs selectively block RNA translation. Currently, one FDA-approved ASO drug, mipomersen, targets apoB and is used in the treatment of familial hypercholesterolemia. AMOs, however, are still in the exploratory stage, and a recent study reported an increment of high-density lipoprotein HDL by the modulation of the ABCA1 gene with miR-33a and miR-33b. Both ASOs and AMOs need further improvements in order to increase their functions and to be more sensitive and specific [155,156]. Other technologies that could show a beneficial therapeutic effect are aptamers, including DNA, RNA or peptide aptamers. Specific aptamers that target pro-inflammatory molecules and the von Willebrand factor, which are associated with endothelial dysfunction, have been created and are undergoing clinical trials [157].

Another potential option is the development of new drugs based on an improvement of natural compounds (phytochemicals) that have been considerably documented to have a positive effect in CVD. Phytochemicals were attributed to attenuate CVD’s main mechanisms of inflammation and oxidative stress. Phytochemicals from fruits (polyphenols: anthocyanins, ellagitannins, catechins, tannins), vegetables (flavonoids: quercetin, lycopene), spices (piperine, safranal) and other sources (polyphenols: catechins, epicatechin, 3-gallate, epic-gallocatechin) have been shown to have cardioprotective effects and to reduce CVD factors [158,159,160,161,162,163].

It is well known that nutrients are crucial drivers of the cellular performance in our bodies. As such, it is not surprising that there is a remarkable interest in the relation between dietary habits and an association with oxidative stress. Even though the exact mechanisms and pathways in which antioxidant nutrients could be involved have not been fully elucidated yet, a link between fruit, vegetables, nuts and dietary patterns has been commonly reported.

Vitamin precursors, such as carotenes, vitamins A, E and C, as well as selenium, zinc, magnesium and resveratrol, are known to have antioxidant properties and can provide protection against oxidative damage and control of the glucose balance [164,165]. Nutritional studies with specific nuts, such as pistachios and almonds, have shown a reduced lipid peroxidation and a protective effect against oxidative stress in subjects with diabetes and metabolic syndrome [166,167].

Several studies have shown that, although some of these antioxidant nutrients can decrease oxidative stress or ameliorate CVDs, unintended detrimental side-effects can occur [168]. Vitamin C intake to reduce oxidative damage has also shown a molecular association with early-onset of obesity [169], and resveratrol has been reported to induce oxidative breakage of DNA in leukaemia [170]. Therefore, a simple nutritional supplementation needs to be carefully balanced to avoid these unwanted secondary effects, but still maintain its prime purpose.

## 6. Conclusions

**Figure 5:**
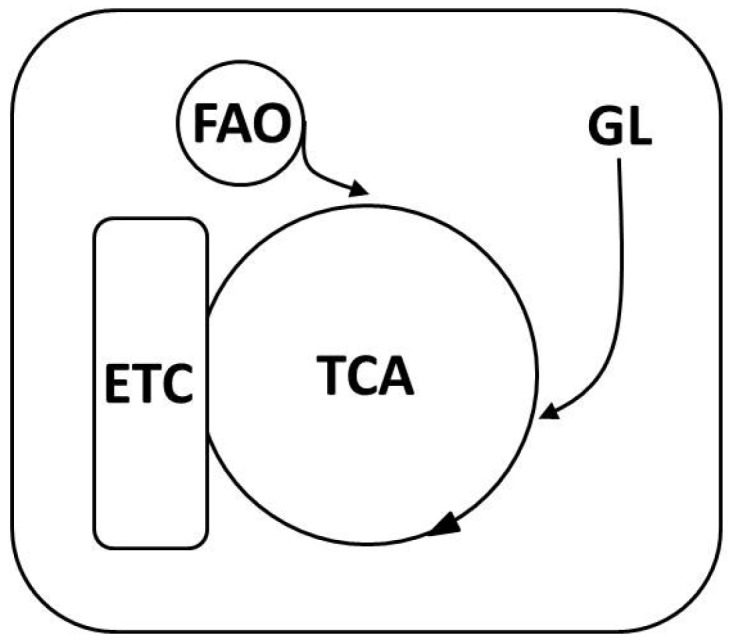
Molecular pathways associated with CVD etiology. Contributing CVD factors, such as diabetes, cause an imbalance of essential physiological pathways by impairing normal glycolysis (GL), which feeds into the tricarboxylic acid pathway (TCA) that is directly linked to the electron transport chain (ETC). Fatty acids, which can also contribute and feed into the TCA cycle through fatty acid oxidation (FAO), are also implicated in CVD manifestation. Multiple enzymes involved in this scheme have been shown to either modulate or directly generate ROS.

Though the influence of oxidative stress in CVD genesis has been clearly elucidated, the main factor that remains is the deciphering of the precise mechanisms involved in CVD pathophysiology. Despite this lack of information, several advances have been made regarding possible targets involved in CVD pathogenesis, such as inflammation, oxidation, adhesion molecules, LDLs, endothelial tissue, leucocytes and other factors. Their disturbance or alteration can potentially lead to endothelial dysfunction, which is the main factor in CVD development. It has also become evident that numerous interlinked cellular metabolic cascades form the basis of CVD and are intricately linked and modulated by ROS (Figure 5).

Over the years, a number of discrepancies and inconsistencies were reported among the different research studies, which to a great extent might be due to different approaches that were used to address research questions, as well as the characteristics of the samples, such as type of subjects, conditions or drug doses. This in turn calls for larger studies to be conducted to ascertain the effect of a specific factor, a better understanding of the molecules involved in the precise stages of CVD development and ultimately the description of more reliable biomarkers that can be targeted and potentially used in the development of novel therapies.

Despite all of the remaining gaps and uncertainties in CVD genesis and development, as well as the associated mechanisms affected by oxidative stress and its specific targets, research in this area is constantly revealing new insights of CVD pathophysiology. Through the implementation of novel integrative approaches, it might be possible to elucidate and decipher, in a concrete way, how the biological systems within the body interact, where exactly damage occurs, where in the cell and at which stage of the disease.

## Figures and Tables

**Table 1:** Cardiokines as potential CVD biomarkers.

Cardiokines	Expression Status	Condition
Atrial and B-Type Natriuretic Peptides	Highly expressed	In rats with pressure overload
	Low level of expression	In mice under fasting conditions
GDF-8	Highly expressed	When skeletal muscle mass is increased
GDF-15	Highly expressed	In patients with cardiac hypertrophy and chronic HF
CTRP9	Low level of expression	In diabetic animals and humans. In subjects that have suffered an acute myocardial infarction
